# VACUTAINER^® ^CPT™ and Ficoll density gradient separation perform equivalently in maintaining the quality and function of PBMC from HIV seropositive blood samples

**DOI:** 10.1186/1471-2172-7-11

**Published:** 2006-05-25

**Authors:** Joyce J Ruitenberg, Candice B Mulder, Vernon C Maino, Alan L Landay, Smita A Ghanekar

**Affiliations:** 1 BD Biosciences, San Jose, CA, USA; 2 RUSH University Medical Center, Chicago, IL, USA

## Abstract

**Background:**

For immune monitoring studies during HIV vaccine clinical trials, whole blood specimens from HIV seropositive (HIV^+^) patients may be collected at multiple sites and sent to a central location for peripheral blood mononuclear cell (PBMC) isolation, cryopreservation and functional evaluation. In this study we show a comparison of two PBMC preparation options, Ficoll density gradient separation (Ficoll) and Cell Preparation Tubes (CPT) using shipped whole blood specimens from 19 HIV^+ ^patients (CD4 > 350, viral load < 50). The pre- and post- cryopreservation performance of samples collected by these two methods were compared by assessment of antigen-specific IFNγ expression in CD8^+ ^and CD8^- ^T cells, cellular viability, and cellular recovery.

**Results:**

The results indicate that cryopreserved PBMC samples tested for CMV- and HIV- specific interferon-gamma (IFNγ) expression performed equivalent to the respective fresh PBMC processed under both collection conditions. Compared to fresh PBMC, the viability was significantly lower for cryopreserved PBMC derived using Ficoll, although it was never less than 90%. There were no significant differences in the IFNγ response, viability, or recovery between cryopreserved PBMC derived by Ficoll and by CPT.

**Conclusion:**

These data suggest that CPT is an efficient system for the collection and cryopreservation of functionally active HIV^+ ^PBMC, as well as a viable alternative to Ficoll gradient separation.

## Background

Therapeutic HIV vaccine clinical trials typically involve the collection of whole blood specimens from HIV^+ ^patients at multiple study sites, and the shipment of collected samples to a central location for PBMC isolation and evaluation [1,2]. Generally, clinical researchers prefer to harvest and cryopreserve PBMC from blood samples collected at pre-determined time points, and perform assays at a later date or after a number of samples have been accumulated. Cryopreservation and the evaluation of samples at a central location have become standard procedures for minimizing operator dependent variability and to improve the precision and accuracy of immunoassays [2,3]. There are two typical whole blood collection options available to clinical investigators, (1) collection in evacuation type/VACUTAINER^® ^tubes and (2) collection in VACUTAINER^® ^CPT™ (Cell Preparation Tube). With collection method (1), samples are collected and shipped to a central location for PBMC isolation using Ficoll density gradient separation, while with collection method (2) whole blood samples are collected, and processed by centrifugation at the collection site, and then shipped to a central location for PBMC recovery.

Previous studies have shown that many factors can have an effect on T cell functional responses including shipment, storage, sample age, cryopreservation, and thawing [1,3,4]. Some of these studies conclude that immunophenotyping, proliferation assays and functional assays should only be done on fresh samples [1,3,4], while others [2] have determined that use of frozen PBMC is a feasible option for monitoring of immune function. Therefore, for studies involving T cell responses as a measure of immune function, it is desirable to know that PBMC samples are processed and handled in a manner that will not degrade the ability of the cells to respond to activation stimuli. It is likewise important that enough viable cells are available after PBMC isolation and/or cryopreservation to perform the desired studies and that the processing method not be technically complex. Compared to CPT-processing, Ficoll density gradient separation is typically a more labor-intensive process requiring an operator with more technical expertise and as such, could result in operator variability with regard to cellular recovery. Thus, to determine if CPT-processed samples can be used as a standard replacement for Ficoll-processed samples, we compared the two PBMC collection and processing methods, using whole blood specimens from 19 HIV^+ ^patients that were shipped to a central location, to ascertain if one method had a significantly different effect on viability, recovery and T lymphocyte responses in antigen specific functional assays than the other method. Since cryopreservation of patient samples has become a standard practice in many clinical trials, the effect of cryopreservation of PBMC collected by both methods was also evaluated in this study.

## Results

**Figure 1:**
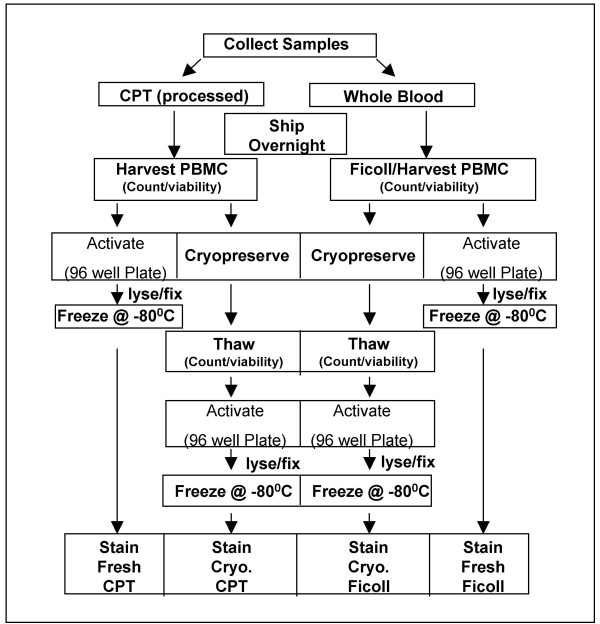
**Study design**. Viability, recovery, and intracellular cytokine staining of activated PBMC were compared for fresh and cryopreserved HIV^+ ^PBMC processed by different methods according to the flow chart depicted here.

To evaluate the effects of CPT and Ficoll processing on HIV^+ ^blood specimens, the study was designed as depicted in the flow chart in Figure 1.

### Cellular viability

**Figure 2:**
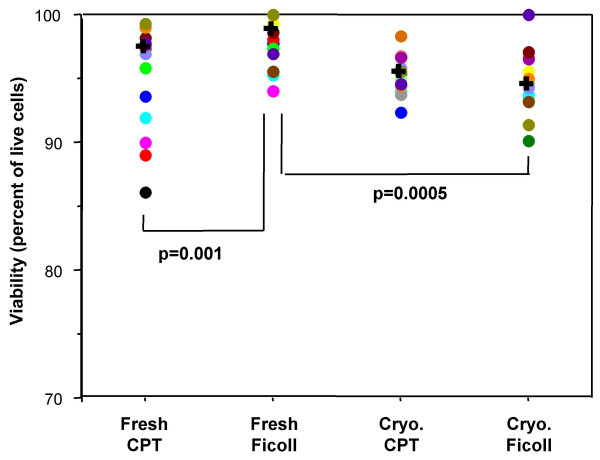
**Viability of CPT-processed PBMC is not adversely affected by cryopreservation**. The viability (percent of live cells) of fresh and cryopreserved PBMC was compared based on processing method. Significant difference (p) was determined by the Wilcoxon signed rank test. Each filled circle represents an individual donor and each donor is represented by the same color for each of the conditions tested. The "**+**" represents the median of all the donors in the category.

Viability of the PBMC was assessed immediately after PBMC preparation (fresh) and again immediately after thawing (cryopreserved). The median viability of Ficoll-processed fresh PBMC was significantly higher than that of Ficoll-processed cryopreserved PBMC (p = 0.0005, Figure 2). There were no significant differences between CPT-processed fresh or cryopreserved PBMC. Viability of Ficoll-processed fresh PBMC was significantly higher compared to CPT-processed fresh PBMC (p = 0.001, Figure 2). The viability of cryopreserved PBMC was always greater than 90% for both processing methods and the differences in viability of the cryopreserved PBMC prepared by either method were not significant.

### Cellular recovery

**Figure 3:**
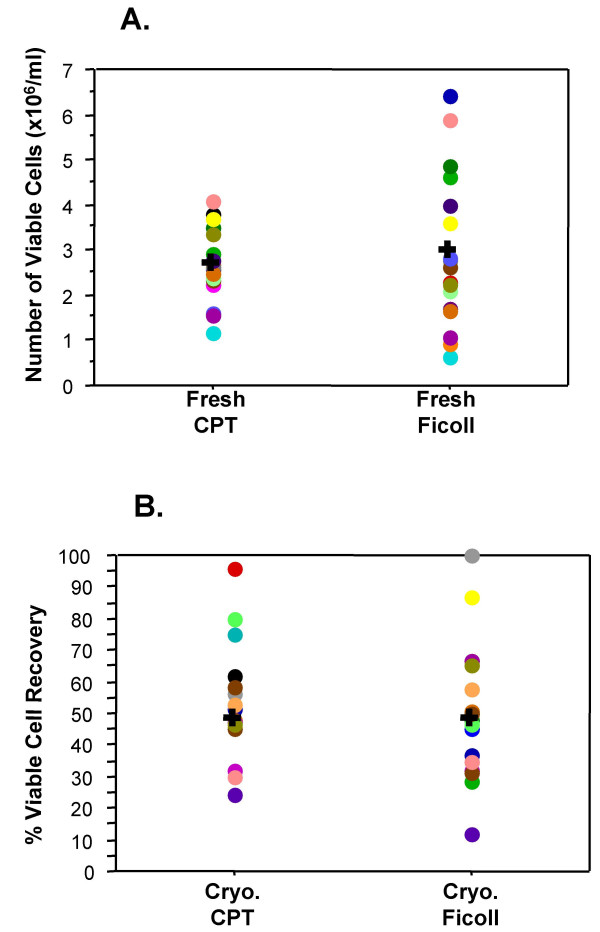
**Generally equivalent PBMC yields are obtained regardless of processing method and no significant differences are seen in cryopreserved PBMC recovery**. The number (cells/ml) of viable "fresh" PBMC recovered after processing are depicted in graph A. Graph B depicts the percentage of thawed, viable PBMC recovered from the number originally frozen down. In these two graphs, each filled circle represents an individual donor and each donor is represented by the same color for each of the conditions tested. The "**+**" represents the median of all the donors in the category. The Wilcoxon signed rank test was used to determine significance.

There were no significant differences between the recoveries (yield of viable cells) of fresh or cryopreserved PBMC processed using Ficoll or CPT. The mean number of viable cells recovered from CPT-processed fresh PBMC was 2.68 × 10^6 ^viable cells/ml of blood and the mean yield of Ficoll-processed fresh PBMC was 2.98 × 10^6 ^viable cells/ml of blood (Figure 3A). The median recovery of viable PBMC after cryopreservation (percent of cells originally cryopreserved) was equivalent for CPT-processed (48.0%) and Ficoll-processed (47.9%) samples (Figure 3B).

### Functional responses to antigen

**Figure 4:**
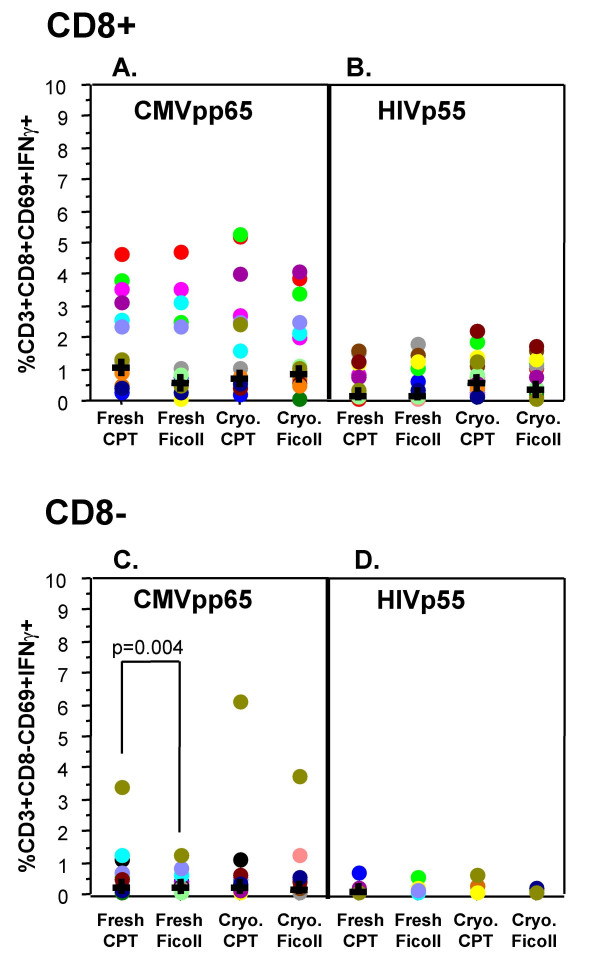
**Processing method does not adversely impact functional response**. IFNγ expression by CD8^+ ^and CD8^- ^T Cells, in response to 6 hour activation with either CMVpp65 peptide mix (A and C) or HIVp55 peptide mix (B and D), was used as a read-out of functional ability of the PBMC. Data displayed have been corrected for the unstimulated expression of cytokine. For FACS analysis, a lymphocyte (forward vs. side scatter) gate, and a CD3+CD8+ gate (A and B) or a CD3+CD8- gate (C and D) were applied. Significant difference (p) was determined by the Wilcoxon signed rank test. Each filled circle represents an individual donor and each donor is represented by the same color for each of the conditions tested. The "**+**" represents the median of all the donors in the category.

The functionality of CD8 positive (CD8^+^) T cells, as measured by IFNγ expression, in fresh or cryopreserved PBMC, processed by both methods was not significantly different when stimulated with CMV-pp65 peptide mix (Figure 4A) or HIV-p55 peptide mix (Figure 4B). The functionality of CD8 negative (CD8^- ^i.e. CD4^+^) T cells was significantly different (p = 0.004) between CPT-processed and Ficoll-processed fresh PBMC for CMV-pp65 peptide mix activation with the overall CPT-processed response being higher than that of the Ficoll-processed. After cryopreservation there was no significant difference between the methods (Figure 4C). There were no significant differences for HIV-p55 peptide mix activation in the fresh or after cryopreservation comparisons within the CD8^- ^group (Figure 4D).

## Discussion

The immunological monitoring of antigen-specific T cell responses is becoming a commonly used assessment tool for therapeutic HIV trials [5] and as such, standardization of sample handling and control of assay variables have become an important necessity. In an effort to minimize operator and inter-assay variability, and improve precision and accuracy, clinical specimens from HIV^+ ^patients are typically collected at different sites and sent to a central location for processing and testing [2,3]. This also facilitates the accumulation and archiving of samples (via cryopreservation) for testing at a future time. Archiving of samples is a desirable study feature, especially when single-cell assays, such as cytokine flow cytometry (intracellular cytokine staining) are used. Staining multiple samples, accumulated over time, from the same donor can potentially minimize within-donor variability, and can also provide a means for avoiding time or operator related staining inconsistencies. Therefore it is desirable to know that samples are handled in a manner that will not compromise the ability of the cells to respond to activation stimuli, that the viability of the cells is not significantly altered, and that enough cells are recovered to perform the desired assays. This study represents an analysis of the effects of PBMC processing method and cryopreservation on viability, recovery and functional response of HIV seropositive whole blood samples. Specimens from 19 HIV^+ ^donors were shipped to a central location for processing and analysis. Since cryopreservation and thawing can have deleterious effects on the viability, recovery and function of PBMC [6-8], a consensus cryopreservation method [9,10] was used for all samples. The same individual performed all PMBC processing (with the exception of the CPT centrifugation step performed at the blood collection site), counting, cryopreservation, functional assays, staining, and analysis to minimize operator variability.

The viability of a sample at the time of cryopreservation will help predict the viability when the sample is thawed. The low viability of a cryopreserved sample can adversely affect *in vitro *functional responses [11]. Disis, et al state that viabilities of > 70% predict consistent responses by T cells in functional studies[10]. It is therefore important that functional assays be performed with samples that are cryopreserved using methods optimized to preserve cellular viability [9-11]. In this study we report that Ficoll-processed fresh PBMC have significantly better viability than CPT-processed fresh PBMC. However, after cryopreservation there was no significant difference between the two methods, indicating that if frozen samples were to be used, both methods would perform equivalently. It is unclear as to why the Ficoll-processed fresh PBMC have a better viability than the CPT-processed fresh PBMC, but since RBC are present only in the shipped specimens that are eventually processed by Ficoll, one could speculate that the RBC presence could be involved in maintaining viability. The fact that the viability is not adversely impacted by cryopreservation and thawing (viability is >90%) indicates that archived samples are acceptable to use in functional studies and should perform equivalently to fresh samples. It should also be noted from the viability results in Figure 2 that, for five CPT processed donors, the viability of the fresh sample was lower than the viability of the thawed cryopreserved sample. One explanation for this could be counting error (either prior to, and/or after cryopreservation), as there is little chance that cellular viability would improve after cryopreservation. Another possible reason could be related to the removal of dead cells from post-thaw washes. Despite this discrepancy, there is no significant difference between the viability of the total fresh and total cryopreserved CPT processed samples.

An important consequence of cryopreservation and thawing, that requires consideration, is diminished cell numbers. The number of cells recovered can be affected by the wash steps involved and by variations with regard to the number of cells originally placed in the freezing vessel. Since it was important to ensure that a sufficient number of PBMC were obtained to complete the comparisons for each processing method, twice as much whole blood was collected for the CPT method as was collected for the Ficoll method. This feature was designed into the study because of reports from other investigators (verbal communications) that CPT-processing routinely yields less PBMC per ml of whole blood than Ficoll-processing yields. As it turned out, the difference in the mean number of viable cells recovered by the Ficoll process (2.98 × 10^6 ^cells/ml of blood) and the CPT method (2.68 × 10^6 ^cells/ml of blood) was not significant (p = 0.3547). In situations where samples are cryopreserved for evaluation at a later date, the numbers of fresh recovered PBMC are important because they determine the number of cells available for freezing and ultimately the post thaw recovery numbers as well. In this study the median post-thaw recovery of viable cells was approximately 50% of the viable cells originally cryopreserved for samples processed by both methods, and we did not see a significant difference between these recoveries. Thus, the PBMC processing method used should not matter when samples are to be used post-cryopreservation. However, considerable numbers of cells were lost (relative to the number originally frozen down) from the majority of donors in this study for both processing methods. The reason for the cell loss was not investigated in this study, but has been reported by other investigators [2,6], and because of this loss it is essential that the investigator collects an excess of sample in order to ensure recovery of sufficient numbers after cryopreservation no matter which processing method is used to obtain the PBMC.

Cytokine Flow Cytometry (CFC) assays can be used to rapidly determine the ability of whole blood or PBMC to respond to antigenic stimuli [12-15]. If the handling or processing of samples has adversely affected a sample, changes in the cytokine response may be evident. The four-color flow cytometry assay employed for this study uses IFNγ expression as a readout for the functional performance of PBMC after shipping, processing and cryopreservation. The comparison of the magnitude of the response to the antigenic stimuli used in this study (CMV-pp65 or HIV-p55 peptide mixes) demonstrate that for the CD3^+^/CD8^+ ^T cells there were no significant differences in response for either processing method, both fresh and after cryopreservation. For the CD3^+^/CD8^- ^T cells, a significant difference was seen between the freshly processed CPT and Ficoll samples in response to CMV-pp65 peptide mix, although the median responses are almost identical (0.21% for CPT and 0.18% for Ficoll). From Figure 4, it appears that this difference may have been due to one "outlier" sample however, subsequent statistical analysis, that excluded the outlier, still gave a significant response (p = 0.006).

For the CFC assays performed in this study, all the functional response data shown are the result of subtracting the background (unstimulated) response. Occasionally, an elevated background response (or spontaneous cytokine production) was observed. In a study of healthy CMV^+ ^donors it was found that spontaneous cytokine production was the result of CMV-responsive cells being reactivated *in-vivo *[16]. In this study it is not known if the unstimulated response is a result of *in-vivo *stimulation, sample manipulation or some other mechanism. For CD3^+^/CD8^+ ^T cells, the backgrounds were generally higher in the samples that were cryopreserved prior to stimulation, with the background of the cryopreserved Ficoll-processed samples being significantly higher (p = 0.002) than the background of the cryopreserved CPT-processed samples (data not shown). The differences in backgrounds of CD8^+ ^T cells between fresh and cryopreserved samples, among all the donors, were not significant for either processing method (data not shown). Similar to CD3^+^/CD8^+ ^T cells, the backgrounds for CD3^+^/CD8^- ^T cells were elevated in the cryopreserved samples prepared by both methods, and an increase in the background of the fresh Ficoll-processed samples was also observed (data not shown). There was a significant difference (p = 0.03) in unstimulated response of CD8^- ^T cells between the fresh and cryopreserved CPT-processed samples (data not shown).

**Figure 5:**
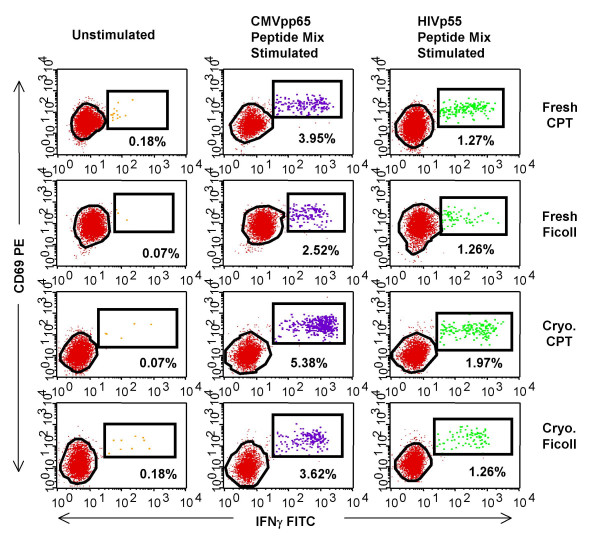
**Antigen-specific stimulation of T cells results in cytokine expression**. Intracellular staining with IFNγ FITC/CD69 PE/CD8 PerCPCy5.5/CD3 APC by Ficoll or CPT-processed PBMC from one representative HIV^+ ^donor. The numbers displayed on the dot-plots represent the percentage of CD3^+ ^and CD8^+ ^lymphocytes that are expressing IFNγ and CD69 in response to antigen-specific stimulation or unstimulated control.

The dot plots in Figure 5 demonstrate IFNγ staining in unstimulated, CMV-pp65 peptide mix- and HIV-p55 peptide mix-stimulated PBMC for one of the donors in this study. It was noted that the staining patterns varied depending on the processing condition and this appeared to be true with all the donors although there was no pattern that could be consistently matched to a particular processing method or activation condition (data not shown). Because of this, it would seem prudent that an experienced technician monitor all analysis in order to insure that the analysis gates are adjusted properly, with the ultimate goal of minimizing a potential source of assay variability[17].

Previous investigators[13] have demonstrated that PBMC and whole blood perform similarly when assessing antigen-specific responses in both CD4+ and CD8+ T cell subsets, with CFC assays, when no sample shipment was involved. However, as discussed above, PBMC are the standard sample used for clinical studies and as such this study was done to compare the PBMC processing methods. It is apparent from the data presented here that CPT-processed PBMC performed equivalently to Ficoll-processed PBMC, especially with regard to the fact that no matter which process was used, samples did not lose functional activity upon cryopreservation and thawing.

## Conclusion

With fresh samples, Ficoll-processed PBMC demonstrated significantly better viability as compared to CPT-processed PBMC. With cryopreserved samples, CPT-processed PBMC demonstrated better viability as compared to Ficoll-processed PBMC but the difference was not significant. Fresh and post-cryopreservation recoveries were not significantly different between the two processing methods. We also conclude that whether fresh or cryopreserved PBMC are used, CPT is an efficient system for the collection of functionally active HIV^+ ^PBMC as there was only one instance (fresh CPT vs. cryopreserved CPT for CD8^- ^T cells) in which the results vary significantly. The results of this comparative study indicate that PBMC prepared by either method perform equivalently and that the use of CPT to prepare PBMC from whole blood could be a viable alternative to using Ficoll density gradient separation when samples are collected at multiple study sites and sent to a central location for processing and evaluation.

## Methods

### Patient characteristics

A total of 19 HIV-seropositive donors with CD4 count greater than 350 and viral loads less than 50 (historically) were recruited by Rush University in Chicago and The Gladstone Institute in San Francisco. All patients were on highly active antiretroviral therapy (HAART) at the time of blood collection.

### Sample collection, processing and handling

Two 8 ml VACUTAINER^® ^CPT™ (Cell Preparation Tube) (BD, Franklin Lakes, NJ) and one 8 ml green top VACUTAINER^® ^(BD, Franklin Lakes, NJ), both containing sodium heparin as the anti-coagulant, were collected from each subject at each site. The CPT were processed at the collection site, according to the manufacturer's instructions, by centrifugation at 1800 × G for 20 minutes at room temperature within two hours of blood collection. After centrifugation the CPT were gently inverted several times. The processed CPT and the whole blood vacutainer were shipped overnight, at ambient temperature, to San Jose, CA. Upon arrival in San Jose, the whole blood from the vacutainer was processed using Ficoll Paque (Amersham Biosciences, Piscataway, NJ) to collect the PBMC. Briefly, whole blood in the vacutainer tube was transferred to a 50 ml conical tube (BD Falcon, Franklin Lakes, NJ), diluted to a volume of 30 ml with HBSS (Gibco Invitrogen Corporation, Grand Island, NY), and underlayed with 10 ml of Ficoll Paque. The 50 ml tubes were centrifuged at 400 × G for 30 minutes after which, the PBMC were collected at the interface layer. PBMC were collected from the processed CPT by gently inverting the collection tube several times and drawing off the PBMC containing plasma with a pipette. PBMC from both sets of tubes were washed twice with HBSS and counted for recovery and viability using 0.4% Trypan Blue (Sigma, St. Louis, MO).

### Cryopreservation of PBMC

Aliquots of PBMC collected by both methods were cryopreserved using a consensus cryopreservation protocol as previously described [9,10]. Briefly, a stock of 2X freezing media containing 20% DMSO in RPMI (Sigma) with 12.5% human serum albumin (HSA, Gemini Bioproducts, Woodland, CA) was prepared and stored at 4°C. At time of use, the 2X freezing media was kept cool on ice. A minimum of 10^7 ^PBMC was frozen in order to ensure sufficient recovery for the performance of functional assays after thawing. Depending on the number of PBMC recovered after processing, the cells were resuspended at a concentration of 1–2 × 10^7^/ml in cold RPMI+12.5% HSA without DMSO. An equal volume of 2X freezing media was added drop wise to the cell suspension with swirling. One ml of cell suspension was distributed to 2 ml cryovials (Sarstedt, Inc., Newton, NC) on ice. Cryovials were then transferred to a freezing container (Nalgene, Rochester, NY), stored at -80°C for 24 hours and then transferred to liquid nitrogen for long-term storage.

### Thawing of PBMC

Upon removal from liquid nitrogen, cryovials were transferred to a 37°C water bath for rapid thawing. One ml of warm (37°C) RPMI+10% fetal bovine serum+antibiotics (cRPMI-10, all components from Sigma) was added drop wise to each thawed cryovial and the total contents of the cryovial were transferred to a 50 ml centrifuge tube (BD Falcon, Bedford, MA) containing 8 ml of warm cRPMI-10. Cells were centrifuged for 7 minutes at 250 × g, resuspended in a small volume of warm cRPMI-10 and counted using Trypan Blue. Cell volume was then adjusted for the appropriate amount needed for stimulation (typically 5 × 10^6^/ml).

### Stimulation of fresh PBMC for CFC assays

If enough cells were available (approximately 10^7^), PBMC were stimulated, immediately after processing as previously described [15,18]. 200 μl of cell suspension in cRPMI-10 was added to each well of a 96-well polypropylene V-bottom plate (BD Falcon) containing activation reagents in a volume of 20 μl. The PBMC were typically distributed at 1 × 10^6 ^cells/well but this concentration ranged between 2 × 10^5 ^and 2 × 10^6^cells/well depending on the donor. CMV-pp65 peptide mix (BD Biosciences, San Jose, CA; 1.7 μg/ml/peptide), HIV-p55 peptide mix (BD Biosciences; 1.7 μg/ml/peptide), and Staphylococcal enterotoxin B (SEB, Sigma; 1 μg/ml) were used as stimuli. All samples received brefeldin A (BFA, BD Biosciences) at a final concentration of 10 μg/ml. An unstimulated control, containing only BFA was also included. The cells were then incubated for 6 hours at 37°C. After 6-hour stimulation at 37°C all samples were held overnight at 18°C using a programmable water bath. Cells were treated with 2 mM EDTA for 15 minutes at room temperature, fixed with 100 μl 1X FACS Lysing Solution (FLS, BD Biosciences) and, after sealing the 96 well plate with parafilm, frozen at -80°C until ready to be stained.

### Stimulation of cryopreserved samples for CFC assays

Frozen PBMC from both processing methods (CPT and Ficoll – processed) were thawed as described above. Cells in each sample were counted to determine viability and recovery, and stimulated as described above for fresh. For all patients, efforts were made to plate the cryopreserved PBMC at the same concentration (cells/well) as their fresh counterpart. After plating, the PBMC were rested overnight in a 37°C, 7% CO_2 _incubator and then stimulated as described above for fresh samples. After stimulation, samples were frozen in FLS at -80°C in the 96 well plate, until ready to be stained.

### Staining of CFC assays

Intracellular staining was performed as previously described [17]. When activated frozen samples from several subjects had been accumulated, the plates were removed from the freezer and thawed at 37°C. Upon thawing, 100 μl of cold wash buffer (PBS/0.5%BSA/0.1%NaN_3_) was added to each well and plates were centrifuged at 500 × G for 5 minutes after which the cells were permeabilized with 200 μl 1X FACS Permeabilizing Solution 2 (BD Biosciences) for 10 minutes and washed twice with 200 μl of wash buffer. Following permeabilization, the samples were stained with a 4-color monoclonal antibody (mAb) cocktail containing IFNγ FITC, CD69 PE, CD8 PerCP-Cy5.5 and CD3 APC (BD Biosciences) for 1 hour at room temperature in the dark. Plates were washed two times and the cells were resuspended in 200 μl of 1% paraformaldehyde in PBS. Stained samples were acquired within 24 hours on a BD FACSCalibur equipped with a High Throughput Sample loader using Multiwell Plate Manager and CellQuest Pro software (BD Biosciences).

### Data analysis

Data on PBMC from CPT and Ficoll samples were compared for viability of fresh and frozen PBMC, recovery of fresh and frozen PBMC, CD8^+ ^and CD8^- ^IFNγ responses to antigenic stimuli, of frozen PBMC. If enough PBMC were available, then analysis for CD8^+ ^and CD8^- ^IFNγ responses was also performed for fresh PBMC. To evaluate functional responses based on intracellular staining, activated samples were compared to unstimulated samples (comparably stained) in order to account for spontaneous cytokine production; hence no isotype control staining was performed. Flow cytometry data were analyzed using CellQuest Pro with automated (snap-to) gating algorithm [15]. Statistical analysis was performed with the Wilcoxon Signed Rank Test (StatView software, SAS Institute Inc., Cary, NC), the non-parametric version of the paired t-test.

## Competing interests statement

The authors, JJR, VCM, and SAG, declare that they are employed by BD, the manufacturer of various materials used in this study including VACUTAINER^® ^CPT™ (Cell Preparation Tube) and that BD is financing the article-processing charge. The authors declare that they have no competing non-financial interests.

## Authors' contributions

JJR wrote the manuscript, the other authors reviewed and edited the manuscript. VCM, ALL and SAG planned and designed the study. SAG supervised the study. CBM and ALL provided donor samples. JJR processed and analyzed the samples, compiled the data, and performed statistical analysis.
